# Identification of *PROK2* gene polymorphisms as predictors of methamphetamine use disorder risk and indicators of craving scale in the Chinese Han population

**DOI:** 10.3389/fphar.2023.1217382

**Published:** 2023-07-06

**Authors:** Zhao Jiang, Tianxiao Zhang, Wei Han, Jing Xiao, Wenpei Zhang, Xiaochen Wang, Jianing Liu, Ying Yang, Congying Yang, Fanglin Guan, Tao Li, John P. Rice

**Affiliations:** ^1^ Department of Forensic Medicine, School of Medicine and Forensics, Xi’an Jiaotong University, Xi’an, Shaanxi, China; ^2^ Key Laboratory of National Health Commission for Forensic Sciences, Xi’an Jiaotong University Health Science Center, Xi’an, China; ^3^ Department of Neurology, Honghui Hospital of Xi’an Jiaotong University, Xi’an, Shaanxi, China; ^4^ Department of Epidemiology and Biostatistics, School of Public Health, Xi’an Jiaotong University, Xi’an, Shaanxi, China; ^5^ Department of Psychiatry, School of Medicine, Washington University in St. Louis, St. Louis, MO, United States

**Keywords:** methamphetamine use disorder, prokineticin 2 gene, genetic polymorphism, craving degree, case-control study

## Abstract

**Background:** Methamphetamine use disorder (MUD) has become a global problem due to the highly addictive nature of methamphetamine. Earlier research have demonstrated that PROK2 functions as a compensatory and protective response against neurotoxic stress by stimulating astrocyte reactivity. The aim of our study was to evaluate the correlation between the *PROK2* gene and both MUD risk susceptibility and craving scale in the Chinese Han population.

**Methods:** A total of 5,282 participants (1,796 MUD patients and 3,486 controls) were recruited. Seven tag SNPs of the *PROK2* gene were chosen and genotyped in the samples. Genetic association analyses were performed to capture the significant SNPs. To investigate the relationship between PROK2 levels and craving scores with the associated-SNP genotypes, we conducted a linear model.

**Results:** SNP rs75433452 was significantly linked with MUD risk (*p*-value = 1.54 × 10^−8^), with the A allele being positively correlated with an increased risk of MUD. Moreover, the average serum level of PROK2 decreased when more copies of the A allele were presented in both MUD patients (*p*-value = 4.57 × 10^−6^) and controls (*p*-value = 1.13 × 10^−5^). Furthermore, the genotypes of SNP rs75433452 were strongly correlated with the craving scores in MUD patients (*p*-value = 4.05 × 10^−4^).

**Conclusion:** Our study identified a significant association signal of the *PROK2* gene with MUD risk susceptibility and methamphetamine craving scores in the Chinese Han population, providing potential valuable insights into the underlying mechanisms of METH dependence.

## Introduction

Methamphetamine (METH), a potent central stimulant and a member of the amphetamine-type drugs class, has emerged as a significant global problem due to its highly addictive nature. Between 2015 and 2016, the use of METH increased fourfold (Piper et al., 2018), and mortality associated with METH use doubled from 2009 to 2019 (Paulus and Stewart, 2020). The detrimental effects of METH use disorder (MUD) extend to multiple organs (Prakash et al., 2017), including the gut, brain, heart, and liver. Users of METH often experience severe anxiety, depression, and even psychosis (Wearne and Cornish, 2018). These symptoms may persist for several years even after discontinuing METH use (Jayanthi et al., 2021). Studies have also shown that prenatal exposure to METH can lead to alterations in the microstructure of the entire brain and changes in subcortical volumes across multiple brain regions in the fetus (Kunkler et al., 2022). A meta-analysis revealed that children exposed to METH prenatally were more likely to exhibit poorer intellectual functioning, problem-solving skills, short-term memory, and language development (Kunkler et al., 2022). The mechanism underlying MUD involves synaptic plasticity in key brain circuits (Kauer and Malenka, 2007), with neurotransmitters such as dopamine (Wise and Robble, 2020), glutamate (Jayanthi et al., 2014), and serotonin (Muller et al., 2007) playing crucial roles. Despite advancements, the precise mechanisms of MUD remain unclear, and currently, there are no effective drugs for its treatment (Morais et al., 2018; Chen et al., 2021).

Recent research has shown that sulforaphane, a potent antioxidant, effectively reduced locomotor activity in mice following acute METH administration and behavioral sensitization (Chen et al., 2012). Nuclear factor erythroid 2-related factor 2 (Nrf2), an essential endogenous factor for combating oxidative stress (Kim and Jeon, 2022), has been implicated in METH-induced toxicity, particularly in dopaminergic neuron endings and DNA oxidation, as evidenced in Nrf2-deficient mice (Nrf2^−/−^). Additionally, icariside II (a phosphodiesterase-5 inhibitor) demonstrated the ability to attenuate METH-induced neurotoxicity and behavioral impairments, which were also associated with oxidative stress (Huang et al., 2022). Notably, Prokineticin 2 has been observed to exhibit a compensatory protective response against neurotoxic stress by inducing astrocyte reactivity in certain experiments (Li et al., 2001). Hence, it is reasonable to speculate that *PROK2* might exert a similar effect in the context of METH-related oxidative stress. Prokineticin (PROK) is a novel group of chemokines discovered in mammals, comprising notable members including Prokineticin 1 (PROK1) and Prokineticin 2 (*PROK2*) (Negri and Ferrara, 2018). These chemokines play crucial roles in diverse physiological processes, including neurogenesis, regulation of circadian rhythms, hematopoiesis, and angiogenesis (Neal et al., 2018). *PROK2* has been identified to be related with emotional-like behaviors (Kishi et al., 2009; Moschetti et al., 2019), suggesting its potential involvement in METH-related anxiety and depression. Although a study conducted in 2011 failed to establish an association between *PROK2* and MUD in the Japanese population (Kishi et al., 2011), it is worth noting that the sample size was limited, which might have contributed to the lack of association. Therefore, further investigation involving a larger sample size is warranted. In the present study, we aimed to address these gaps by conducting association analyses using a substantial sample size, aiming to evaluate the relationship between the *PROK2* gene and both susceptibility to MUD and the craving scale in Han Chinese individuals.

## Methods

### Power analysis

We utilized the genetic power calculator (https://zzz.bwh.harvard.edu/gpc/) to calculate the minimum sample size required to achieve the desired level of statistical power. Supplementary Table S1 provides a summary of the parameters used for the power analysis. Our results indicate that to achieve 80% statistical power, we need to recruit 1,878 and 1,775 MUD patients for genotypic and allelic analysis, respectively. In the present study, we have enrolled 1,796 MUD patients and this sample size could achieve 77.58% and 80.57% statistical power for genotypic and allelic analysis, respectively. In general, our current sample size level could provide enough statistical power for detecting SNPs with significant effects.

### Study subjects

The participants were all Han Chinese individuals who were not linked genetically. The study participants were limited to the Han Chinese population in order to control, at least to some extent, population stratification, which is a significant confounding factor in gene association mapping studies. The patients were enrolled at Chang’an Drug Rehabilitation Center (Xi’an city, Shaanxi). Based on the Diagnostic and Statistical Manual of Mental Disorders, Fifth Edition (DSM-V), study participants were classified as METH dependent if they had substance use disorders and had used the drug at least twice a week for over a year. Individuals who satisfied any of the following conditions were not included in the study: 1) addiction disorders (as defined by DSM-V for other addictive substances); 2) presence of tumors; 3) neurodegenerative conditions; 4) severe organic disorders; and 5) any history or current diagnosis of psychotic disorders. The healthy control group was enrolled at the Second Affiliated Hospital of Xi’an Jiaotong University, and they were verified to be free of personal or family history of drug use disorders or psychotic disorders through clinical evaluation. Each participant’s peripheral blood was sampled for genotyping. The enzyme-linked immunosorbent assay (ELISA) kits from Life Span BioSciences, Inc. (Seattle, WA, United States) were utilized to assess PROK2 levels in the serum, following the instructions provided with the kits. Demographic data was gathered via questionnaires. For each MUD patient, the METH usage year, average dose, and craving scores were obtained. To measure craving scores, visual analog scales (VAS) were employed. All participants gave their consent in writing after being fully informed. The study procedures were approved by the Medical Ethics Committee of the Xi’an Jiaotong University Health Science Center. More information about the study subjects could be obtained from the previous publication of our research team (Wang et al., 2022).

### SNP selection and genotyping

Candidate SNPs were selected based on genetic information coverage. Initially, we retrieved all the *PROK2* gene region SNPs with a minor allele frequency (MAF) of 0.01 or higher (based on 1,000 Genomes CHB data). A total of 22 SNPs were obtained. Then tag SNPs were generated based on the criteria of *r* ^2^ > 0.8. The tagging algorithm employed in this study was proposed by de Bakker et al. (2005) It offers a combination of the simplicity seen in pairwise *r* ^2^ methods with the potential efficiency found in multimarker haplotype approaches. Finally, 7 SNPs were chosen and genotyped in the samples. Commercial DNA kits were used to extract genomic DNA from all participants (Axygen Scientific, Inc., Union City, California, United States). The Sequenom MassARRAY platform was used to conduct SNP genotyping. Those technicians involved in the experiments were not aware of the labels on the study samples. To ensure quality control, around 5% of the study samples were selected at random for replication of SNP genotyping, resulting in a 100% concordance rate.

### Statistical analysis

Minor allele frequencies were calculated for each genotyped SNP. To ensure quality control, Hardy-Weinberg equilibrium (HWE) tests were performed on all genotyped SNPs to assess their conformity with the HWE. Genetic association analyses were carried out at the individual marker level. The SNPs were coded in four genetic modes. In the genotypic model, individuals were assigned values of 0, 1, or 2 corresponding to the presence of 0, 1, or 2 copies of the minor allele. For the recessive model, individuals were assigned a value of 0 if they had 0 or 1 copy of the minor allele, and a value of 1 only if they had two copies of the minor allele. In the dominant model, individuals were assigned a value of 0 if they had 0 copies of the minor allele, and a value of 1 if they had 1 or 2 copies of the minor allele. In addition, allelic analysis was also conducted to compare the allelic distribution of SNPs between MUD patients and controls. Genetic association analysis was performed using Plink software (version 1.9) (Purcell et al., 2007). Haploview software (version 4.2) was used to visualize the patterns of linkage disequilibrium (LD) (Barrett et al., 2005). To address the issue of multiple comparisons, Bonferroni corrections were applied.

To explore the potential functional implications of the selected SNPs, the GTEx database was utilized to extract *PROK2* gene expression data (Consortium, 2013). The impact of the selected SNP on the expression of the *PROK2* gene was then summarized and evaluated in multiple human tissues. Serum levels of PROK2 were also compared among individuals with different genotypes in both MUD patients and controls. The craving scores of MUD patients were compared among individuals with different genotypes of the targeted SNPs using a linear model. General statistical analyses were performed using R software (version 4.2.2), with age, gender, length of METH use, and average dose of METH use included as covariates.

## Results

### Descriptive statistics

A total of 5,282 individuals (1,796 individuals with MUD and 3,486 controls) were enrolled (Table 1). There were no significant variations observed in terms of age (*t* = 0.11, *p*-value = 0.91) and gender (*χ* ^2^ = 0.01, *p*-value = 0.93) between the MUD patients and the controls. The two groups exhibited noteworthy differences in terms of employment (χ2 = 998.09, *p*-value < 0.01) and marital status (χ2 = 694.88, *p*-value < 0.01). Generally, a higher percentage of MUD patients were found to be unemployed and single or divorced, as compared to the control group. Moreover, the mean serum levels of PROK2 protein were significantly elevated in MUD patients in comparison to controls (*t* = 267.64, *p*-value < 0.01). For MUD patients, the average length of METH use is 4.8 years. The average dose of METH used is 0.6 g. The average craving scores for the MUD patients were 3.4.

**TABLE 1:** The demographic and characteristic information of the study subjects.

Variables	MUD patients (N = 1,796)	Controls (N = 3,486)	t/χ^2^	*p*-value
Age (years), mean ± sd	34.9 ± 8.5	34.9 ± 9.3	0.1075	0.91
Gender (%)				
*Male*	1,164 (65)	2,265 (65)		
*Female*	632 (35)	1,221 (35)	0.0077	0.93
Employment status (%)				
*Employed*	946 (53)	3,166 (91)		
*Unemployed*	850 (47)	320 (9)	998.09	<0.01
Marital status (%)				
*Single or Divorced*	1,115 (62)	870 (25)		
*Married*	681 (38)	2,616 (75)	694.88	<0.01
Serum level of PROK2(pg/mL), mean ± sd	3826.7 ± 245.3	2036.4 ± 197.9	267.64	<0.01
Length of METH use (years), mean ± sd	4.8 ± 1.6	-	-	-
Average dose of METH use (g), mean ± sd	0.6 ± 0.3	-	-	-
Craving scores, mean ± sd	3.4 ± 1.5	-	-	-

### Genetic association of rs75433452 genotypes with MUD

**FIGURE 1:**
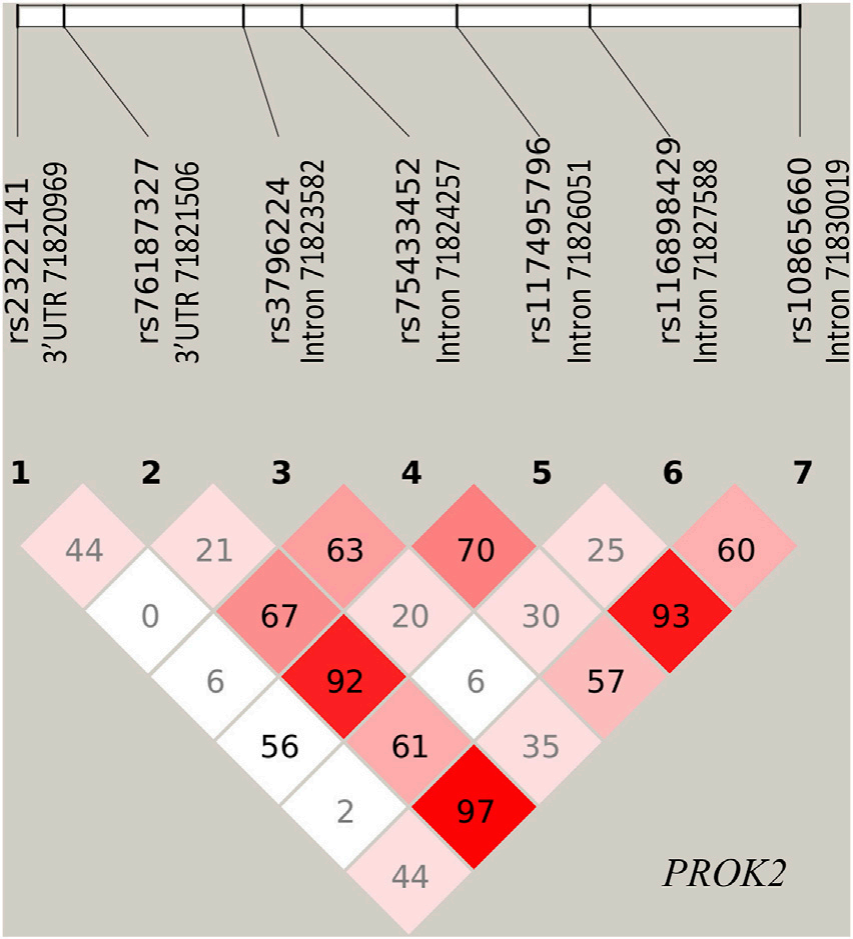
The Linkage disequilibrium plot for the genotyped SNPs in gene *PROK2*. Values of D′ were indicated in each cell.

Supplementary Table S2 reported the *p*-value in controls for each genotyped SNP tested for HWE. Figure 1 indicated the LD plot of the genotyped SNPs, wherein no LD blocks were identified. None of the seven SNPs showed any significant deviation from HWE. Among them, only SNP rs75433452 was strongly associated with MUD risk in all four genetic modes (Table 2; Supplementary Table S3). A significant correlation was observed between the A allele of this SNP and an increased risk of MUD [OR (95%CI) = 1.27 (1.17–1.37)]. A dose-dependence pattern was also observed in the genotypic analysis. Individuals with the AA genotype had an OR (95%CI) of 1.64 (1.38–1.94) and those with the AG genotype had an OR (95%CI) of 1.22 (1.38–1.94), as compared to individuals with the GG genotype.

**TABLE 2:** The significant association between genotypes of SNP rs75433452 and METH dependence.

SNP	Modes	Groups	MUD patients (N = 1,796)	Controls (N = 3,486)	χ^2^	OR (95%CI)	*p*-value
rs75433452	ALLELIC	A	1,566 (44)	2,643 (38)		1.27 (1.17–1.37)	
		G	2,026 (56)	4,329 (62)	32.0	ref	1.54 × 10^−8^
	GENO	AA	343 (19)	485 (14)		1.64 (1.38–1.94)	
		AG	880 (49)	1,673 (48)		1.22 (1.07–1.38)	
		GG	573 (32)	1,328 (38)	33.2	ref	6.17 × 10^−8^
	REC	AA	343 (19)	485 (14)		1.46 (1.25–1.70)	
		AG + GG	1,453 (81)	3,001 (86)	24.1	ref	9.10 × 10^−7^
	DOM	AA + AG	1,223 (68)	2,158 (62)		1.31 (1.16–1.48)	
		GG	573 (32)	1,328 (38)	19.7	ref	8.96 × 10^−6^

ALLELIC, allelic mode; GENO, genotypic mode; REC, recessive mode; DOM, dominant mode.

The threshold of *p* values were 0.05/7 ≈ 0.007.

### Functional consequences of SNP rs75433452

**FIGURE 2:**
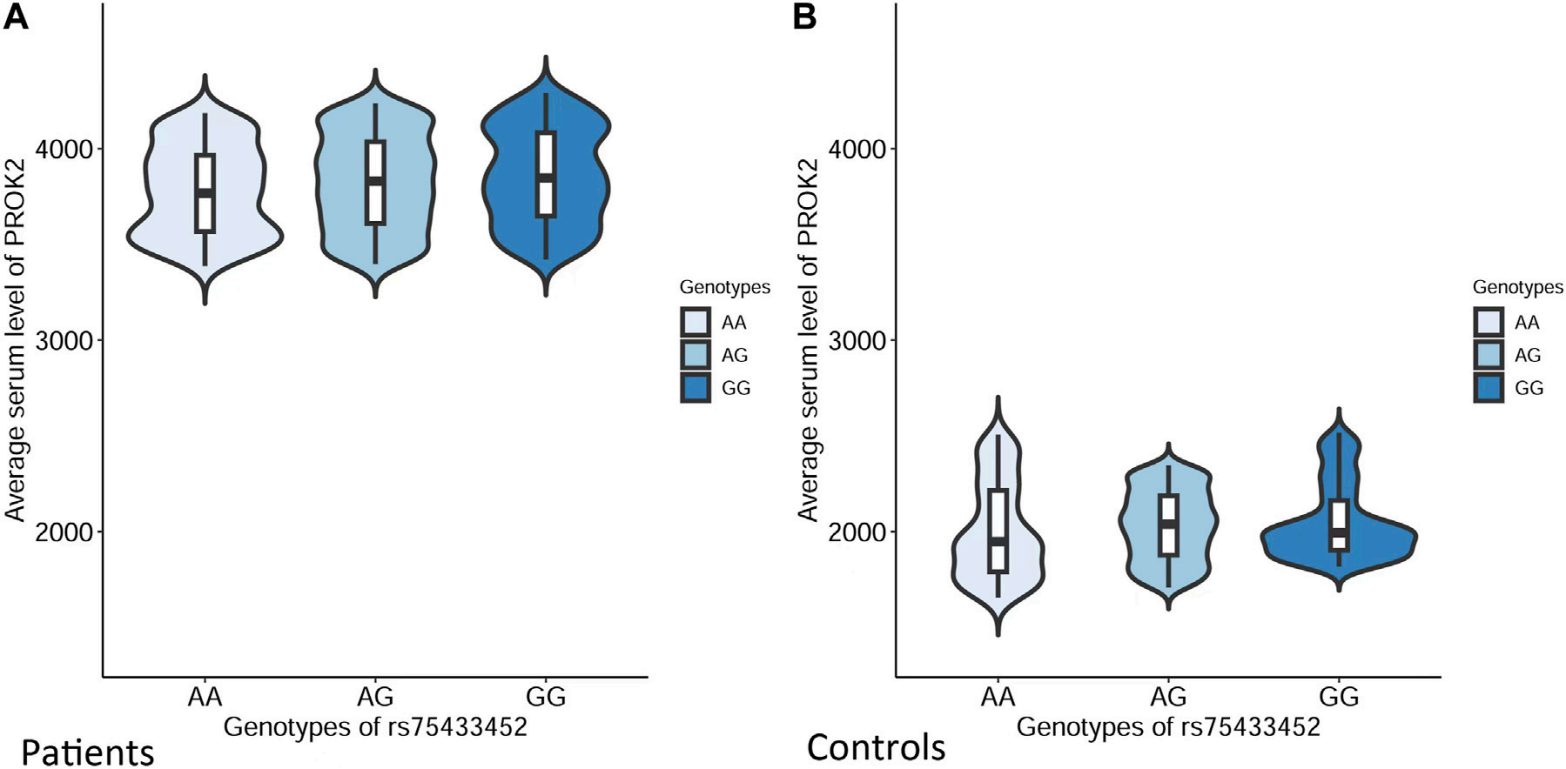
Average serum levels of *PROK2* in different genotypic groups of SNP rs75433452 in both MUD patients and controls. The standard deviation was indicated by the error bar. Results of analysis of variance in MUD patients **(A)**: *F* = 21.14, *p*-value = 4.57 × 10^−6^; Controls **(B)**: *F* = 19.34, *p*-value = 1.13 × 10^−5^.

Using gene expression data from multiple human tissues obtained from GTEx, we compiled the potential expression quantitative trait loci (eQTL) signals for SNP rs75433452 with respect to the *PROK2* gene. However, no significant signals were obtained (Supplementary Table S4). Notably, significant differences in average serum level of PROK2 were identified among individuals with different rs75433452 genotypes in both MUD patients and controls (Figure 2). The average serum level of *PROK2* decreased when more copies of the A allele were presented in MUD patients (*F* = 21.14, *p-*value = 4.57 × 10^−6^). This pattern was not observed in controls although significant differences were also identified for average serum level of *PROK2* among different genotype groups (*F* = 19.34, *p-*value = 1.13 × 10^−5^).

### Association between SNP rs75433452 and craving score in MUD patients

The genotypes of SNP rs75433452 were strongly correlated with the craving scores in MUD patients (Table 3). On average, one more copy of the minor allele (A allele) would increase the craving scores of 0.18 (*t* = 3.54, *p-*value = 4.05 × 10^−4^). This effect was survived even if covariates including age, gender, length of METH use, and average dose of METH use were adjusted.

**TABLE 3:** Relationship between craving scores and genotypes of SNP rs75433452.

SNP	Genotypes	Craving scores	*β*	*t*-Statistics	*p*-value	*^a^P-*adjusted
rs75433452	AA (N = 828)	3.58 ± 1.53	0.18	3.54	4.05 × 10^−4^	3.93 × 10^−4^
	AG (N = 2,553)	3.38 ± 1.56				
	GG (N = 1,901)	3.21 ± 1.49				

^a^ *p*-value was adjusted for genotypes of rs75433452 in the linear model including age, gender, length of METH, use, and average dose of METH, use as covariates.

## Discussion

The current study has discovered a genetic variation (SNP rs75433452) within the *PROK2* gene that is associated with the susceptibility to MUD risk in large-scale individuals with Chinese Han ancestry. The findings of our study have also indicated a significant correlation between this SNP and the serum levels of *PROK2* protein. Additionally, in patients with MUD, this SNP is found to be linked with craving scores. A study conducted by Kishi *et al* failed to show a significant connection between the *PROK2* gene and MUD in Japanese populations (Kishi et al., 2011). Four SNPs were genotyped and analyzed in their study and two of them were also included in our study (rs10865660 and rs3796224). The inconsistent findings between the present study and the previous study could potentially be attributed to variations in sample size and genetic marker coverage between the two studies. To the best of our knowledge, this study is the first to establish a link between MUD risk and genetic variations in the *PROK2* gene within the Han Chinese population.

SNP rs75433452 is an intronic DNA variant of *PROK2*. Its functional consequence is still not clear. Despite the significant correlation between this SNP and the serum level of *PROK2* protein observed in our study, data obtained from the GTEx suggest that this SNP does not exhibit a significant correlation with the *PROK2* expression in any of the 48 tissues. In this sense, there is still a missing link in the chain of evidence. Nevertheless, evidence from the publicly available database might be suffered from low quality and high heterogeneity resulting from a small sample size. Another major limitation of the GTEx database is that the clinical information of samples are unknown. As the patterns of gene expression are significantly influenced by the sample status, the gene expression patterns obtained from normal samples may be quite different from samples of MUD patients.

Many pieces of evidence show that oxidative damage is existing in MUD. Above-normal potentially toxic lipid peroxidation products appeared in the brains of chronic users of METH (Toborek et al., 2013). The administration of METH was found to result in a marked elevation of superoxide radicals in the brain capillaries of mice, as evidenced by DHE staining (Sajja et al., 2016). As known, the blood-brain barrier (BBB) comprises of crucial components such as the endothelial cells of the brain and astrocytes. The oxidative stress induced by METH was found to result in damage to the endothelial cells and astrocytes (Northrop and Yamamoto, 2012), ultimately leading to the impairment of the BBB. This is considered to be one of the most significant events associated with METH toxicity (Davidson et al., 2001). *PROK2* and its receptor, *PROKR2*, play an essential role both in physiological conditions and neuropathological processes (Koyama et al., 2006). Interestingly, a recent GWAS based on European populations has linked *PROK2* to smoking initiation. SNP rs116516927 within the PROK2 gene was found to exhibit a significant association with the initiation of smoking (Saunders et al., 2022). In the central nervous system (CNS), *PROKR2* is mostly expressed in neurons, whereas *PROK2* is mainly expressed in astrocytes and microglia, indicating their complex and diverse biological functions (Koyama et al., 2006; Maftei et al., 2014). The expression of *PROK2* increased under pathological insults, such as hypoxia and ROS, indicating that it is involved in these cellular responses (Lattanzi et al., 2021). Through the stimulation of mitochondrial biogenesis and activation of the ERK and Akt survival signaling pathways, the elevated expression of the *PROK2* gene facilitated a compensatory protective response in Parkinson’s disease (PD) models and PD brains (Gordon et al., 2016). Based on these previous reports on *PROK2* functions we hypothesize that the pathological mechanisms of *PROK2* on MUD might be similar to these neuropathological processes. In general, the increased expression of *PROK2* is the result of MUD, in turn, which would mediate a compensatory protective response for MUD. Data from the present study could support our hypothesis. The serum levels of *PROK2* in MUD patients are much higher compared to controls, and the A allele related to the increased risk of MUD is also associated with lower serum levels of *PROK2*. Furthermore, the A allele is also strongly correlated with increased craving scores in MUD patients.

Notably, there were several limitations to this study. Firstly, being a candidate gene-based study, the present study only focused on a specific gene, *PROK2*. However, MUD is a complex trait influenced by multiple genes. Secondly, the study did not account for the influence of environmental factors on the development of MUD. The omission of environmental factors in the current study may potentially limit the comprehensiveness of the findings, as environmental factors often interact with genetic factors. Furthermore, it should be noted that the findings of this study may primarily pertain to the Han population, and their generalizability to other ethnic groups with distinct cultural and anthropological traits could be limited. Validation studies based on other ethnic groups are needed in future. Lastly, although we have shown that the serum level of *PROK2* is related to the genotype of rs75433452, no significant results were obtained for the mRNA level. Moreover, the study did not investigate the functional role of the identified SNP in the pathogenesis of MUD. Thus, more complex regulatory signals at the level of related RNAs were not involved in our study. This also led to the lack of systematic analysis of the molecular mechanism behind our results, which is crucial for understanding the complex mechanism of the *PROK2* gene involved in meth addiction. Therefore, in future studies, validation of our results and elucidation of complex mechanisms are needed.

To conclude, our study established a significant association between the *PROK2* gene and the risk of MUD in a large-scale Han Chinese population. It was also found that the A allele of SNP rs75433452 is correlated with the increased risk of MUD and higher craving scores in MUD patients. Our study has yielded potential valuable insights into the underlying mechanisms of METH dependence. By shedding light on this complex process in the future, our findings could pave the way for the development of new and effective treatments for METH dependence.

## Data Availability

The data presented in the study are deposited in the China National GeneBank (CNGB) repository, accession number CNP0004348.
