# Supplementary material for: Identification of PROK2 gene polymorphisms as predictors of methamphetamine use disorder risk and indicators of craving scale in the Chinese Han population
Source: Front Pharmacol. 2023 Jul 6;14:1217382. doi: 10.3389/fphar.2023.1217382 (PMC10356980; doi:10.3389/fphar.2023.1217382)
Supplement: Supplementary file 1 [file Table1.DOCX]

**Supplementary Tables**

Supplementary Table S1. Parameters utilized in the power analysis and sample size estimation.

| Parameters | Values | Note |
| --- | --- | --- |
| High risk allele frequency | 0.17 | Average MAF of the 7 genotyped SNPs |
| Prevalence | 0.03 | reference #2 |
| Gneotype relative risk Aa | 1.25 | Typical effect size of a SNP |
| Gneotype relative risk Aa | 1.25 |  |
| D-prime | 1 |  |
| Marker allele frequency | 0.17 | Average MAF of the 7 genotyped SNPs |
| Number of cases | 1796 | Number of cases enrolled in this study |
| Contorls: case ratio | 1.94 | controls: cases ratio of this study |
| Type I error rate | 0.007 | 0.05/7≈0.007 |
| Statistical power | 0.8 |  |

Supplementary Table S2. Genetic information of the selected SNPs.

| CHR | POS | SNP | FUNC | A1 | A2 | MAF | HWE |
| --- | --- | --- | --- | --- | --- | --- | --- |
| 3 | 71820969 | rs2322141 | untranslated-3 | T | C | 0.07 | 0.59 |
| 3 | 71821506 | rs76187327 | untranslated-3 | C | G | 0.13 | 0.70 |
| 3 | 71823582 | rs3796224 | intron | T | C | 0.23 | 0.47 |
| 3 | 71824257 | rs75433452 | intron | A | G | 0.40 | 0.26 |
| 3 | 71826051 | rs117495796 | intron | C | T | 0.05 | 0.45 |
| 3 | 71827588 | rs116898429 | intron | C | T | 0.04 | 0.33 |
| 3 | 71830019 | rs10865660 | intron | G | A | 0.31 | 0.20 |

CHR: chromosome; POS:position; FUNC: function; A1: minor allele; A2: major allele; MAF: minor allele frequency; HWE: *P* values of the Hardy Weinberg equilibrium test.

Supplementary Table S3. Full results of the single marker based association analyses.

| CHR | SNP | A1 | A2 | Mode | AFF | UNAFF | χ^2^ | DF | *P* Values |
| --- | --- | --- | --- | --- | --- | --- | --- | --- | --- |
| 3 | rs10865660 | G | A | ALLELIC | 1082/2510 | 2155/4817 | 0.6906 | 1 | 0.406 |
| 3 | rs10865660 | G | A | DOM | 911/885 | 1806/1680 | 0.557 | 1 | 0.4555 |
| 3 | rs10865660 | G | A | REC | 171/1625 | 349/3137 | 0.3211 | 1 | 0.571 |
| 3 | rs10865660 | G | A | GENO | 171/740/885 | 349/1457/1680 | 0.6762 | 2 | 0.7131 |
| 3 | rs116898429 | C | T | ALLELIC | 150/3442 | 258/6714 | 1.443 | 1 | 0.2296 |
| 3 | rs116898429 | C | T | DOM | 145/1651 | 251/3235 | 1.303 | 1 | 0.2536 |
| 3 | rs116898429 | C | T | GENO | 5/140/1651 | 7/244/3235 | 1.444 | 2 | 0.4858 |
| 3 | rs116898429 | C | T | REC | 5/1791 | 7/3479 | 0.3148 | 1 | 0.5747 |
| 3 | rs117495796 | C | T | REC | 7/1789 | 10/3476 | 0.3912 | 1 | 0.5317 |
| 3 | rs117495796 | C | T | ALLELIC | 179/3413 | 334/6638 | 0.1905 | 1 | 0.6625 |
| 3 | rs117495796 | C | T | DOM | 172/1624 | 324/3162 | 0.1112 | 1 | 0.7388 |
| 3 | rs117495796 | C | T | GENO | 7/165/1624 | 10/314/3162 | 0.4425 | 2 | 0.8015 |
| 3 | rs2322141 | T | C | DOM | 223/1573 | 457/3029 | 0.5077 | 1 | 0.4762 |
| 3 | rs2322141 | T | C | ALLELIC | 234/3358 | 475/6497 | 0.3373 | 1 | 0.5614 |
| 3 | rs2322141 | T | C | GENO | 11/212/1573 | 18/439/3029 | 0.8639 | 2 | 0.6493 |
| 3 | rs2322141 | T | C | REC | 11/1785 | 18/3468 | 0.2006 | 1 | 0.6543 |
| 3 | rs3796224 | T | C | ALLELIC | 783/2809 | 1604/5368 | 1.977 | 1 | 0.1597 |
| 3 | rs3796224 | T | C | DOM | 693/1103 | 1412/2074 | 1.821 | 1 | 0.1772 |
| 3 | rs3796224 | T | C | GENO | 90/603/1103 | 192/1220/2074 | 1.968 | 2 | 0.3738 |
| 3 | rs3796224 | T | C | REC | 90/1706 | 192/3294 | 0.5784 | 1 | 0.4469 |
| 3 | rs75433452 | A | G | ALLELIC | 1566/2026 | 2643/4329 | 32 | 1 | 1.54E-08 |
| 3 | rs75433452 | A | G | GENO | 343/880/573 | 485/1673/1328 | 33.2 | 2 | 6.17E-08 |
| 3 | rs75433452 | A | G | REC | 343/1453 | 485/3001 | 24.11 | 1 | 9.10E-07 |
| 3 | rs75433452 | A | G | DOM | 1223/573 | 2158/1328 | 19.72 | 1 | 8.96E-06 |
| 3 | rs76187327 | C | G | ALLELIC | 459/3133 | 865/6107 | 0.2987 | 1 | 0.5847 |
| 3 | rs76187327 | C | G | REC | 32/1764 | 56/3430 | 0.2224 | 1 | 0.6372 |
| 3 | rs76187327 | C | G | DOM | 427/1369 | 809/2677 | 0.2133 | 1 | 0.6442 |
| 3 | rs76187327 | C | G | GENO | 32/395/1369 | 56/753/2677 | 0.3526 | 2 | 0.8383 |

CHR: chromosome; POS: position; A1: minor allele; A2: major allele; ALLELIC: allelic mode; GENO: genotypic mode; REC: recessive mode; DOM: dominant mode; AFF: Number of METH dependents; UNAFF: number of controls.

Supplementary Table S4. Effects of SNP rs75433452 on gene expression of *PROK2*.

| Gene | SNP | *P* Values | NES | T-statistic | Tissue |
| --- | --- | --- | --- | --- | --- |
| *PROK2* | rs75433452 | 0.01 | -0.410 | -2.700 | Brain - Hippocampus |
| *PROK2* | rs75433452 | 0.01 | -0.070 | -2.600 | Whole Blood |
| *PROK2* | rs75433452 | 0.05 | -0.380 | -2.000 | Brain - Amygdala |
| *PROK2* | rs75433452 | 0.05 | -0.120 | -1.900 | Muscle - Skeletal |
| *PROK2* | rs75433452 | 0.07 | -0.200 | -1.800 | Brain - Nucleus accumbens (basal ganglia) |
| *PROK2* | rs75433452 | 0.10 | -0.170 | -1.700 | Liver |
| *PROK2* | rs75433452 | 0.11 | 0.150 | 1.600 | Pituitary |
| *PROK2* | rs75433452 | 0.13 | -0.230 | -1.500 | Vagina |
| *PROK2* | rs75433452 | 0.14 | -0.095 | -1.500 | Artery - Tibial |
| *PROK2* | rs75433452 | 0.16 | -0.200 | -1.400 | Cells - EBV-transformed lymphocytes |
| *PROK2* | rs75433452 | 0.17 | -0.100 | -1.400 | Heart - Atrial Appendage |
| *PROK2* | rs75433452 | 0.20 | -0.190 | -1.300 | Brain - Hypothalamus |
| *PROK2* | rs75433452 | 0.23 | -0.230 | -1.200 | Brain - Spinal cord (cervical c-1) |
| *PROK2* | rs75433452 | 0.27 | -0.120 | -1.100 | Artery - Coronary |
| *PROK2* | rs75433452 | 0.28 | -0.083 | -1.100 | Colon - Transverse |
| *PROK2* | rs75433452 | 0.29 | -0.070 | -1.100 | Heart - Left Ventricle |
| *PROK2* | rs75433452 | 0.31 | -0.120 | -1.000 | Adrenal Gland |
| *PROK2* | rs75433452 | 0.32 | -0.061 | -0.990 | Adipose - Subcutaneous |
| *PROK2* | rs75433452 | 0.36 | -0.190 | -0.920 | Brain - Substantia nigra |
| *PROK2* | rs75433452 | 0.37 | 0.072 | 0.900 | Breast - Mammary Tissue |
| *PROK2* | rs75433452 | 0.42 | -0.063 | -0.810 | Skin - Sun Exposed (Lower leg) |
| *PROK2* | rs75433452 | 0.43 | -0.059 | -0.790 | Nerve - Tibial |
| *PROK2* | rs75433452 | 0.45 | -0.120 | -0.760 | Uterus |
| *PROK2* | rs75433452 | 0.46 | 0.110 | 0.750 | Ovary |
| *PROK2* | rs75433452 | 0.49 | -0.085 | -0.690 | Small Intestine - Terminal Ileum |
| *PROK2* | rs75433452 | 0.50 | -0.050 | -0.670 | Artery - Aorta |
| *PROK2* | rs75433452 | 0.54 | -0.085 | -0.610 | Minor Salivary Gland |
| *PROK2* | rs75433452 | 0.57 | 0.065 | 0.570 | Brain - Caudate (basal ganglia) |
| *PROK2* | rs75433452 | 0.57 | -0.044 | -0.560 | Stomach |
| *PROK2* | rs75433452 | 0.62 | -0.071 | -0.490 | Brain - Putamen (basal ganglia) |
| *PROK2* | rs75433452 | 0.65 | -0.020 | -0.450 | Adipose - Visceral (Omentum) |
| *PROK2* | rs75433452 | 0.67 | -0.030 | -0.430 | Spleen |
| *PROK2* | rs75433452 | 0.69 | -0.053 | -0.390 | Brain - Cortex |
| *PROK2* | rs75433452 | 0.69 | -0.038 | -0.400 | Colon - Sigmoid |
| *PROK2* | rs75433452 | 0.69 | -0.026 | -0.400 | Esophagus - Mucosa |
| *PROK2* | rs75433452 | 0.69 | -0.032 | -0.400 | Esophagus - Muscularis |
| *PROK2* | rs75433452 | 0.71 | -0.056 | -0.380 | Brain - Cerebellum |
| *PROK2* | rs75433452 | 0.72 | 0.056 | 0.350 | Brain - Cerebellar Hemisphere |
| *PROK2* | rs75433452 | 0.72 | 0.058 | 0.370 | Brain - Frontal Cortex (BA9) |
| *PROK2* | rs75433452 | 0.73 | -0.028 | -0.350 | Skin - Not Sun Exposed (Suprapubic) |
| *PROK2* | rs75433452 | 0.78 | 0.015 | 0.280 | Testis |
| *PROK2* | rs75433452 | 0.79 | -0.027 | -0.270 | Pancreas |
| *PROK2* | rs75433452 | 0.84 | 0.032 | 0.200 | Brain - Anterior cingulate cortex (BA24) |
| *PROK2* | rs75433452 | 0.89 | 0.006 | 0.140 | Lung |
| *PROK2* | rs75433452 | 0.93 | 0.010 | 0.082 | Prostate |
| *PROK2* | rs75433452 | 0.99 | 0.000 | 0.007 | Thyroid |

NES: normalized effect size.
